# Association of the Endothelial Activation and Stress Index (EASIX) and the sFlt-1/PlGF Ratio in Patients with Preeclampsia

**DOI:** 10.3390/diagnostics16172863

**Published:** 2026-09-06

**Authors:** Anna Sophie Scholz, Alexandra von Au, Anne Marshall, Kristina Killinger, Walid Shaalan, Michael Elsässer, Thomas Luft, Cahit Birdir, Herbert Fluhr, Julia Spratte, Hannah Arndt

**Affiliations:** 1 Department of Gynecology and Obstetrics, Heidelberg University Hospital, 69120 Heidelberg, Germany; 2 Department of Endocrinology, Heidelberg University Hospital, 69120 Heidelberg, Germany; 3 Department Medicine V, Hematology, Oncology, Rheumatology, Heidelberg University Hospital, 69120 Heidelberg, Germany; 4 Department of Gynecology and Obstetrics, Graz University Hospital, 8036 Graz, Austria

**Keywords:** endothelial dysfunction, preeclampsia, EASIX

## Abstract

**Background**: Preeclampsia is a leading cause of maternal and perinatal morbidity, characterized by angiogenic imbalance reflected in the sFlt-1/PlGF ratio. While the Endothelial Activation and Stress Index (EASIX), a composite score of lactate dehydrogenase, creatinine, and platelet count, has recently been proposed as an independent predictor of adverse events in preeclampsia, it has not yet been evaluated against the established sFlt-1/PlGF ratio. This post hoc analysis of a prospective observational trial aimed to characterize the association between EASIX and sFlt-1/PlGF and to compare the prognostic performance of both markers in predicting adverse events. **Methods**: Thirty-two pregnant women diagnosed with preeclampsia were prospectively enrolled at the University of Heidelberg between 2015 and 2017. EASIX was calculated retrospectively from routine laboratory parameters. Spearman correlations, ROC analyses, and Kaplan–Meier curves were applied. **Results**: EASIX correlated significantly with the sFlt-1/PlGF ratio (r = 0.41, *p* = 0.019). While EASIX was not associated with mean uterine pulsatility index (PI) (r = 0.07, *p* = 0.74) or the cerebro–placental ratio, it showed a non-significant trend toward a positive association with mean arterial pressure (r = 0.35, *p* = 0.058). In contrast, sFlt-1/PlGF showed a moderate correlation with mean uterine PI (r = 0.53, *p* = 0.009). Both EASIX (AUC 0.73) and sFlt-1/PlGF (AUC 0.78) showed numerically similar AUC point estimates for adverse maternal events, while sFlt-1/PlGF showed a numerically higher AUC than EASIX for perinatal outcomes (AUC 0.80 vs. 0.66). Elevated EASIX was associated with an increased risk of imminent delivery (HR 3.00), although this estimate is limited by the sample size. **Conclusions**: EASIX and the sFlt-1/PlGF ratio were moderately correlated and were both associated with a shorter time to delivery. The sFlt-1/PlGF ratio showed a numerically higher AUC for predicting perinatal outcomes, while AUC point estimates for maternal outcomes were numerically similar for EASIX and sFlt-1/PlGF ratio.

## 1. Introduction

Preeclampsia remains a leading cause of maternal and perinatal morbidity and mortality [1] and encompasses a heterogeneous spectrum of end-organ manifestations ranging from kidney injury, liver failure, HELLP syndrome, placental insufficiency, and intrauterine growth restriction associated with substantial maternal and fetal compromise [2]. Pregnancy has been conceptualized as a stress test for the maternal endothelium, unmasking subclinical vascular and cardiac vulnerability that may otherwise remain silent [3]. Beyond the acute peripartum period, vascular changes triggered by preeclampsia, including endothelial dysfunction [4] and left ventricular remodeling [5,6], persist postpartum and are associated with a substantially increased long-term risk of cardiovascular and renal morbidity [7,8,9].

Preeclampsia is characterized by an angiogenic imbalance with excessive release of antiangiogenic soluble fms-like tyrosine kinase-1 (sFlt-1) that captures circulating placental growth factor (PlGF) [10], leading to systemic manifestations and impaired vascular and endothelial function [11,12]. The sFlt-1/PlGF ratio has been validated as a predictive marker to rule out the development of preeclampsia in patients with suspected disease [13,14]. There is also evidence that the sFlt-1/PlGF ratio performs well in predicting adverse events in patients with established preeclampsia [15,16,17]. Despite its clinical utility, widespread implementation of the sFlt-1/PlGF ratio remains constrained by substantial assay costs of up to EUR 80 and limited availability outside specialized centers. As a result, reliable risk stratification tools are often lacking in primary care settings, rural regions, and low-resource countries, where emergency access to this biomarker may be particularly restricted.

In contrast, the Endothelial Activation and Stress Index (EASIX) is a composite score consisting of standard laboratory parameters such as lactate dehydrogenase (LDH), creatinine, and platelet counts, and it reflects features of serious endothelial complications such as transplant-associated thrombotic microangiopathy [18]. The endothelial nature of EASIX was further supported by a prospective study that demonstrated associations between EASIX and several endothelial parameters, including glycocalyx thickness, digital perfusion, vascular permeability, and platelet aggregation [19]. As endothelial dysfunction often affects kidney dysfunction, causes cell damage leading to LDH release, and provokes platelet aggregation and consumption due to endothelial cell activation, EASIX uniquely summarizes the key pathophysiological aspect of endothelial dysfunction. Subsequent studies validated EASIX as a prognostic marker in a range of clinical settings, including metastatic pancreatic cancer [20], acute decompensated heart failure [21], and coronary heart disease [22]. Recently, our group introduced EASIX as a novel, independent predictor of maternal adverse events in patients with preeclampsia [23]. Furthermore, EASIX diverged over time with steeper slopes in patients who developed adverse maternal outcomes [24] and reflected disease severity [25]. However, data to evaluate the relationship of EASIX to the sFlt-1/PlGF ratio were not available in these previous studies. Whether EASIX reflects a distinct or overlapping pathophysiological dimension, and whether it offers diagnostic value beyond the sFlt-1/PlGF ratio, has remained unaddressed until now.

As EASIX and the sFlt-1/PlGF ratio have not previously been directly compared, this post hoc analysis of a prospective observational trial was designed as a first, exploratory, hypothesis-generating comparison of these two markers and their relationship with maternal blood pressure and sonographic markers of fetal and uteroplacental perfusion. To give a broader picture of a possible clinical use of EASIX and the sFlt-1/PlGF ratio, we additionally assessed the prognostic performance of EASIX compared to the sFlt-1/PlGF ratio for the time to delivery and development of adverse maternal and perinatal events.

## 2. Materials and Methods

### 2.1. Study Design and Population

This study was a post hoc secondary analysis of a prospectively collected observational cohort. Between October of 2015 and February of 2017, a total of 32 pregnant women diagnosed with either early-onset or late-onset preeclampsia were prospectively enrolled during their treatment at the Department of Obstetrics and Gynecology of the University of Heidelberg. Inclusion occurred at the time of diagnosis of preeclampsia. At the time of prospective recruitment, preeclampsia was diagnosed according to the diagnostic criteria at that time, including a mean blood pressure above 140/90 mmHg in a 24 h blood pressure reading and a confirmed proteinuria above 0.3 g/d in a 24 h urine collection or a protein–creatinine ratio above 30 g/molKr. All patients also fulfilled the subsequently published diagnostic criteria of the International Society for the Study of Hypertension in Pregnancy 2021 guidelines [2]. The study protocol was approved by the Institutional Ethics Committee of the University of Heidelberg, and all participants provided informed consent (S-199/2015). The study protocol conformed to the principles of the Declaration of Helsinki. An amendment for this post hoc analysis was granted in January 2026. Inclusion criteria were defined as pregnancy above 20 weeks, given informed consent and the diagnosis of preeclampsia. Exclusion criteria were pre-existing kidney disease or gestational diabetes requiring insulin therapy, as the initial study protocol aimed to analyze the influence of preeclampsia on kidney function.

### 2.2. Clinical Assessment and Data Collection

Upon admission, all patients underwent a standardized clinical workup including a detailed medical history and a comprehensive fetomaternal ultrasound examination performed within the prenatal diagnostics department. Venous blood samples were collected on the day of study inclusion to perform routine laboratory screening, including liver enzymes, platelet count, and hemolysis markers, and to determine the sFlt-1/PlGF ratio. For the measurement of the sFlt-1/PlGF ratio, a serum sample was collected on the day of study enrollment. After centrifugation, serum was pipetted and stored at −20 °C. After all samples had been collected, sFlt-1 and PlGF were determined using an assay kit from Roche (Elecsys^®^, Roche Diagnostics GmbH, Mannheim, Germany) and were performed by the central clinical laboratory at the University of Heidelberg. Additionally, 24 h blood pressure monitoring was conducted on the day before study enrollment, and mean arterial blood pressure was calculated as follows: (2 × diastolic blood pressure + systolic blood pressure)/3. Screening for proteinuria was assessed via the protein–creatinine ratio (PCR) and 24 h urine protein. Treating clinicians had access to routine laboratory parameters, including creatinine, LDH, and platelet count, but were blinded to the sFlt-1/PlGF ratio. Clinical management followed local institutional guidelines during the study period, which prioritized pregnancy prolongation where clinically feasible. Indications for delivery are summarized in Supplemental Table S1.

### 2.3. Post Hoc Calculation of EASIX

Following the initial prospective phase, a retrospective analysis was conducted on the established dataset. The primary objective was to evaluate the EASIX score in the context of preeclampsia diagnostics. The EASIX score was calculated post hoc using the prospectively collected laboratory parameters as follows: (creatinine (mg/dL) × lactate dehydrogenase (U/L))/platelet count (10^9^ cells per L).

### 2.4. Outcome Measure

Adverse maternal and perinatal events were defined according to the core outcomes set of preeclampsia [26,27]. In detail, maternal adverse events were defined as maternal mortality, cerebrovascular events like eclampsia and stroke, pulmonary edema, raised liver enzymes, acute kidney injury according to the Kidney Disease Improving Global Outcomes definition (increase in creatinine of ≥0.3 mg/dL [≥26.5 μmoL/L] within 48 h, an increase of ≥1.5 times from baseline within 7 days, or urine volume < 0.5 mL/kg per hour for 6 h), postpartum hemorrhage, placental abruption, and the hemolysis, elevated liver enzymes, low platelets syndrome defined as increased transaminases more than twice the upper reference interval with platelets < 100 cells/nL with at least one hemolysis criterion (LDH ≥ 2 times the upper reference limit or indirect bilirubin > 1.2 mg/dL or haptoglobin < 0.3 g/L). Perinatal events included stillbirth, gestational age at delivery below 34 weeks due to preeclampsia, small-for-gestational-age infant defined as birth weight below the 10th percentile, neonatal mortality, seizures, respiratory support and admission to a neonatal unit. In multiple pregnancies, perinatal outcomes were assessed at the pregnancy level. The composite outcome was considered present if it occurred in either neonate.

### 2.5. Statistical Analysis

Data were analyzed to examine the prognostic performance of the EASIX score in comparison to the sFlt-1/PlGF ratio. Given the exploratory nature of this post hoc analysis, no formal sample size calculation was performed. Continuous variables were presented as median (interquartile range). Statistical significance was defined as *p* < 0.05. Correlations were analyzed using the Spearman correlation coefficient for those parameters available from routine measurements and scans. For sonographic parameters in twin pregnancies, the values corresponding to the smaller fetus were used. Detailed sonographic evaluation was not part of the original study protocol and was therefore not available for all patients. Given the small sample size, missing data were handled using available-case analysis without imputations. To assess the prognostic performance of both EASIX and the sFlt-1/PlGF ratio for the development of adverse events, we calculated receiver operating characteristic (ROC) curves and the corresponding area under the curve with 95% confidence intervals. Kaplan–Meier curves were calculated for EASIX and sFlt-1/PlGF to compare the remaining time until delivery. Given the absence of an established EASIX threshold in preeclampsia, we selected a percentile-based cut-off using a consistent approach across both biomarkers: the lowest quartile and tertile of the cohort’s sFlt-1/PlGF distribution (72 and 107, respectively) were compared against the established sFlt-1/PlGF threshold of 85, and the closer-corresponding percentile (25th) was subsequently applied to EASIX (0.599). This threshold is used exclusively for exploratory visualization of the Kaplan–Meier curves and carries no clinical interpretation. The sFlt-1/PlGF threshold of 85 was applied uniformly across the cohort; given the sample size and predominance of early-onset disease, subgroup-specific thresholds were not considered. Kaplan–Meier curves were compared using the log-rank (Mantel–Cox) test, and hazard ratios (HRs) with 95% confidence intervals were estimated using the Mantel–Haenszel method. To account for potential confounding by gestational age at study inclusion, we additionally performed Cox proportional-hazards regression models using gestational age as the underlying time scale (left-truncated Cox model: entry at gestational age at study inclusion and exit at gestational age at delivery). EASIX and the sFlt-1/PlGF ratio were included as continuous variables in the models, with gestational age at inclusion as a covariate. The proportional hazards assumption was assessed using Schoenfeld residuals. Statistical analyses were conducted in Prism 9.5.0 (GraphPad Prism Software, San Diego, CA, USA; Python (v3.10.11), lifelines (pandas v2.3.2, lifelines v0.30.0) [28]; R (v4.6.1), survival package (v3.8.6), accessed via rpy2 v3.6.7). Coding assistance for implementation of the Cox models was provided by Claude (Anthropic, San Francisco, CA, USA); all code, model outputs, and statistical interpretations were reviewed and verified by the authors.

## 3. Results

### 3.1. Study Population

The study population included 32 patients with preeclampsia (Table 1). Most patients had early-onset preeclampsia (*n* = 25), defined as a diagnosis of preeclampsia before 34 weeks of gestation. Median gestational age was 32.5 weeks at study inclusion (range 20.9–36.7) and 33.7 weeks at delivery. Baseline characteristics of the cohort are shown in Table 1. Although inclusion of multiples might increase heterogeneity, comparison of neither EASIX (0.82 IQR (0.52–2.51) vs. 0.80 IQR (0.60–1.05), *p* = 0.62) nor the sFlt-1/PlGF ratio (65.0 IQR (50.5–192.8) vs. 248.6 IQR (119.6–479.7), *p* = 0.068) in patients with multiples versus singleton pregnancies reached the level of significance, possibly attributable to the small subgroup. The composite of maternal adverse events occurred in nine (28%) patients with preeclampsia. The most common outcomes were raised liver enzymes (*n* = 5) and acute kidney injury (*n* = 4). There were two patients with HELLP syndrome, one with pulmonary edema and one with postpartum hemorrhage. Two patients experienced more than one outcome event. The composite of adverse perinatal events occurred in 23 patients (72%), a rate substantially higher than in unselected populations, reflecting the predominance of early-onset PE (*n* = 25, 78%) in this cohort. Thus, the rate of small-for-gestational-age babies (19) and preeclampsia-related preterm delivery before 34 weeks (14) was very high. Ventilatory support for respiratory distress syndrome was needed in six cases. In our cohort no necrotizing enterocolitis, intraventricular hemorrhage, seizures or maternal or neonatal death occurred. However, there were three stillbirths (Supplemental Table S2).

### 3.2. Association Between EASIX and Other Biomarkers

EASIX showed a significant moderate positive correlation with the sFlt-1/PlGF ratio (r = 0.41, 95% CI (0.07 to 0.67), *p* = 0.019) (Table 2). When analyzing the individual EASIX parameters, both LDH (r = 0.44, 95% CI (0.10–0.69), *p* = 0.012) and creatinine (r = 0.47, 95% CI (0.14–0.71), *p* = 0.006) were significantly correlated with sFlt-1/PlGF. When evaluating correlations of all five parameters (LDH, creatinine, platelets, sFlt-1, and PlGF) among each other, only creatinine and PlGF showed a significant inverse association (r = −0.42, 95% CI (−0.68 to −0.06), *p* = 0.02). EASIX was not significantly correlated with gestational age at measurement (r = −0.3, 95%CI (−0.59–0.05), *p* = 0.09), whereas sFlt-1/PlGF was significantly associated with gestational age (r = −0.67, 95%CI (−0.83–−0.42), *p* < 0.0001).

**Figure 1:**
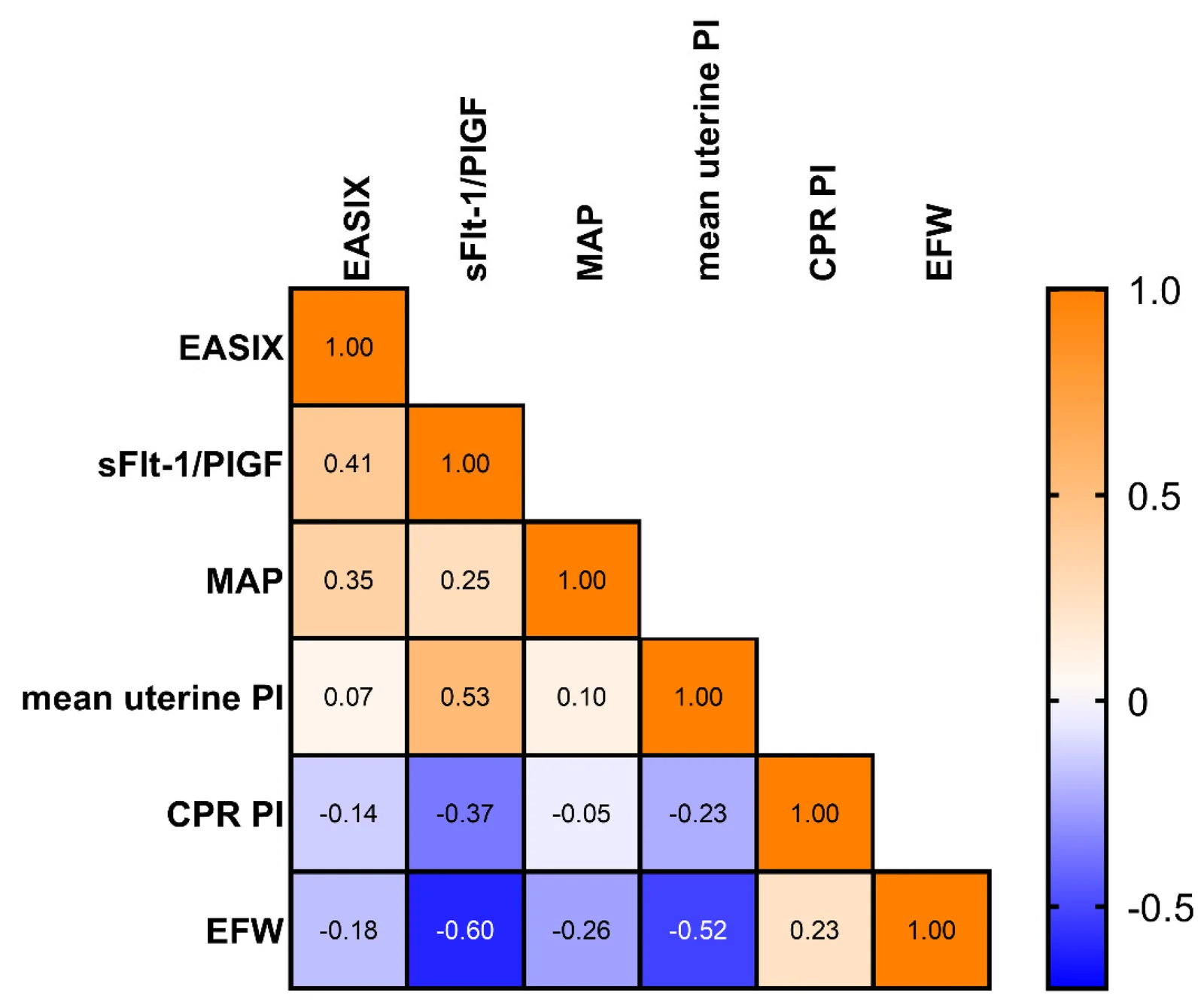
Relationship between biochemical, biophysical and sonographic markers. MAP: mean arterial blood pressure (*n* = 29); PI: pulsatility index; EFW: estimated fetal weight (*n* = 31) calculated according to Hadlock formula; CPR: cerebro–placental ratio (*n* = 28), calculated as the ratio of the middle cerebral artery pulsatility index to the umbilical artery pulsatility index (MCA-PI/UA-PI). Mean uterine artery PI was available for 23 patients and was measured according to the International Society of Ultrasound in Obstetrics and Gynecology (ISUOG) practice guidelines [29] and was calculated as the average of right and left uterine artery PI. Results should be viewed as exploratory due to small sample size and multiple testing.

In the context of biophysical parameters, EASIX showed a weak to moderate positive correlation with MAP (r = 0.35, 95% CI (−0.02 to 0.64), *p* = 0.058) (Figure 1, Supplemental Table S3). Although this correlation did not reach statistical significance, the confidence interval spans from no association to a moderate–strong correlation, which may reflect insufficient statistical power rather than a true absence of association. EASIX showed little correlation with mean uterine PI (r = 0.07, 95% CI (−0.36 to 0.48), *p* = 0.74). In contrast, mean uterine PI and sFlt-1/PlGF showed a moderate to strong correlation (r = 0.53, 95% CI (0.14 to 0.78), *p* = 0.009).

The cerebro–placental ratio (CPR) PI revealed a weak negative correlation with EASIX (r = −0.14, 95% CI (−0.51 to 0.28), *p* = 0.508) with a wide confidence interval, limiting the precision of this estimate. CPR PI showed a moderate negative correlation with sFlt-1/PlGF (r = −0.37, 95% CI (−0.67 to 0.03), *p* = 0.064). The strongest association was observed for sFlt-1/PlGF and the estimated fetal weight (r = −0.6, 95% CI (−0.79 to −0.30), *p* < 0.001).

### 3.3. Time to Delivery Analyses

**Figure 2:**
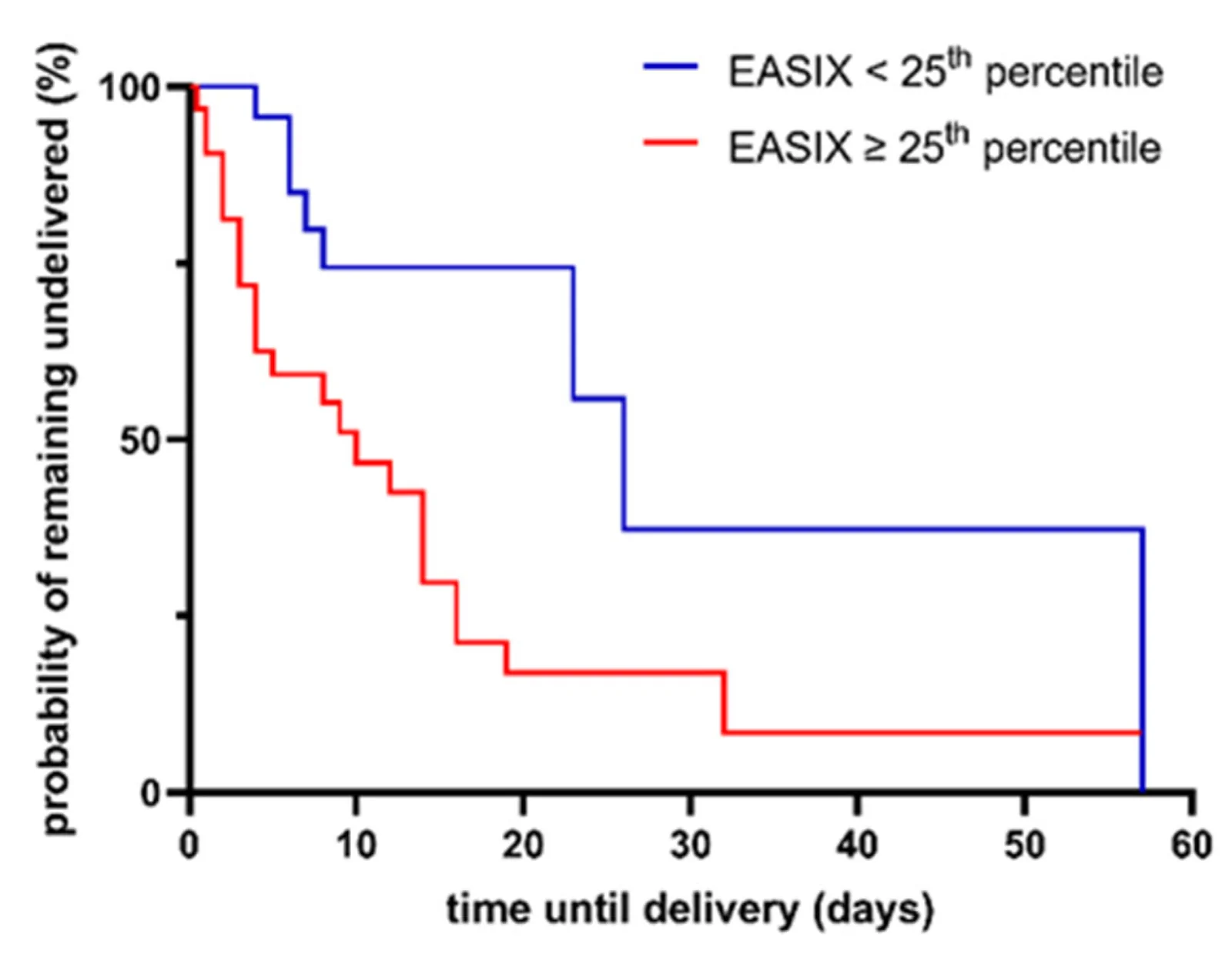
Time to delivery analyses of EASIX. The group of patients with EASIX above the 25th percentile (0.599) included 24 patients and is indicated by the red curve.

**Figure 3:**
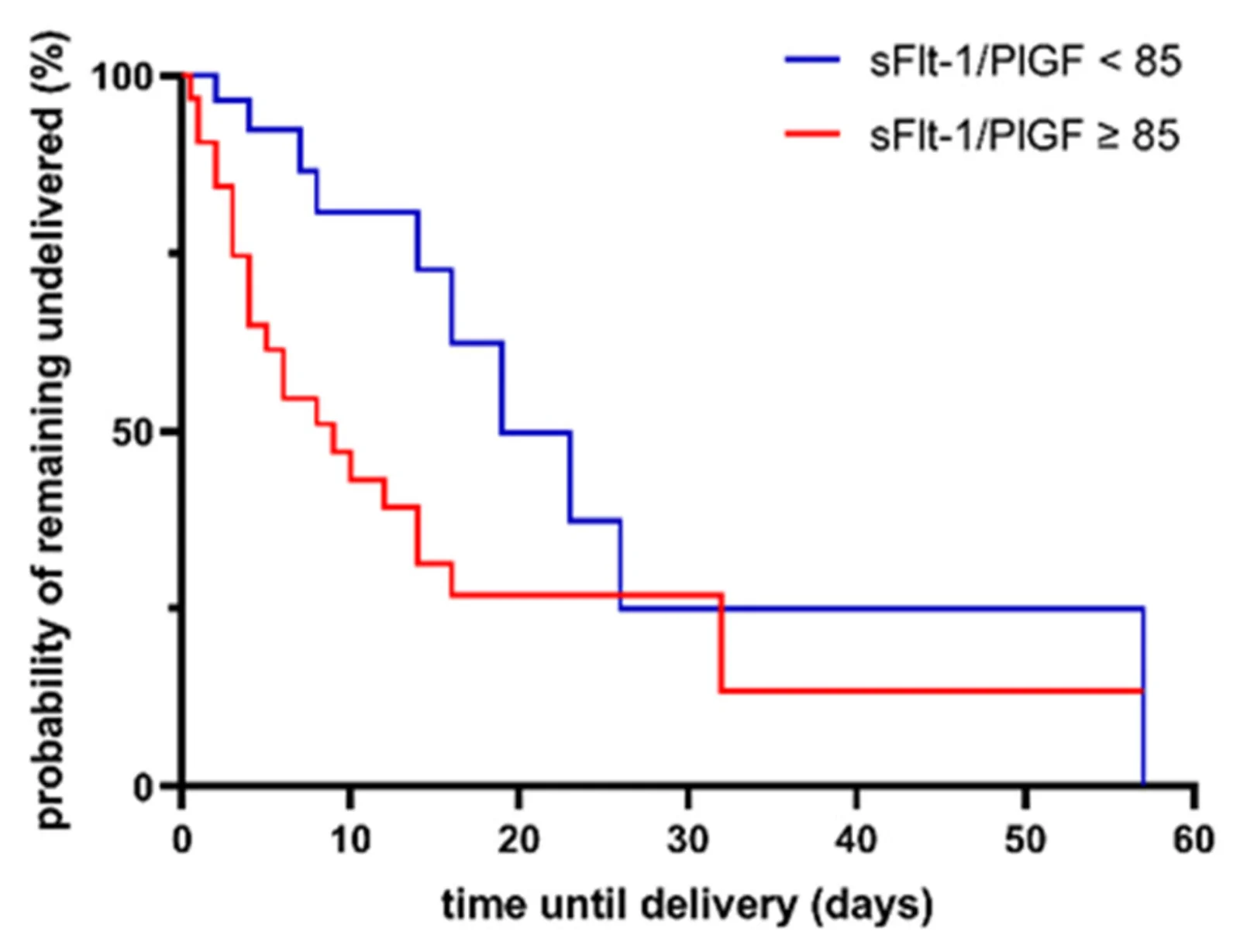
Time to delivery analyses of the sFlt-1/PlGF ratio. The group of patients with a sFlt-1/PlGF ratio ≥ 85 included 22 patients and is indicated by the red curve.

The median time from study inclusion to delivery was 10 days overall, and comparable between patients with early-onset (10.9 days) and late-onset preeclampsia (9.0 days). For exploratory time to delivery analyses, the cohort was dichotomized according to the 25th percentile of EASIX (0.599) and the threshold of 85 for the sFlt-1/PlGF ratio. The threshold of EASIX was not derived for diagnostic or therapeutic use. Patients with EASIX values above the 25th percentile delivered earlier and were at a 3-fold increased risk of imminent delivery (HR 3.00, 95% CI 1.5–6, log-rank *p* = 0.004) (Figure 2). Patients with high sFlt-1/PlGF ratios revealed a hazard estimate of 2.2 (95% CI 1.1–4.4, log-rank *p* = 0.03) for the risk of imminent delivery (Figure 3).

In Cox proportional-hazards models adjusted using gestational age as the time scale, EASIX (continuous) remained significantly associated with the hazard of delivery (aHR 3.09, 95% CI 1.08–8.84). The sFlt-1/PlGF ratio also remained significantly associated with the hazard of delivery (aHR 1.01, 95% CI 1.002–1.01). These hazard ratios represent the relative change in the hazard of delivery associated with a one-unit increase in the respective biomarker and therefore should not be directly compared as measures of effect size because the biomarkers have different numerical scales. The proportional-hazards assumption was assessed using Schoenfeld residuals and was not violated for any covariate in either model (all *p* > 0.4) (Table 3).

### 3.4. Performance of EASIX and sFlt-1/PlGF in Predicting Adverse Events

The composite of adverse maternal events was observed in 28% of the patients. Both EASIX and sFlt-1/PlGF were significantly higher in patients experiencing adverse maternal events (Table 4). Both EASIX (area under the curve (AUC) 0.73, 95% CI 0.53–0.93) and sFlt-1/PlGF (AUC 0.78, 95% CI 0.61–0.94) showed numerically similar point estimates for discrimination of adverse maternal events. The wide and overlapping confidence intervals indicate substantial uncertainty around these estimates and do not support formal comparison of prognostic performance. In a sensitivity analysis restricting the maternal composite to components independent of EASIX variables (excluding acute kidney injury and HELLP syndrome; *n* = 5 events), EASIX showed an AUC of 0.65 (95% CI 0.37–0.93) without statistical significance. The composite of adverse perinatal events was diagnosed in 72% of patients with preeclampsia, primarily owing to the high rate of early-onset preeclampsia. The sFlt-1/PlGF ratio was significantly higher in patients who developed adverse perinatal events (Table 4). While EASIX did not demonstrate statistically reliable discrimination for the development of adverse perinatal events (AUC 0.66, 95% CI 0.41–0.91), sFlt-1/PlGF yielded an area under the curve of 0.80 (95% CI 0.64–0.95).

## 4. Discussion

This post hoc analysis of a prospective trial provides the first characterization of EASIX in relation to the sFlt-1/PlGF ratio, evaluating the prognostic capacity of both biomarkers side-by-side in patients with preeclampsia. We found that EASIX was moderately correlated with the sFlt-1/PlGF ratio, and that both markers showed distinct association patterns with biophysical and sonographic parameters, suggesting that they capture different pathophysiological aspects of preeclampsia. In addition, our data suggest an increased risk of imminent delivery and adverse maternal events in patients with higher EASIX levels.

The moderate, statistically significant correlation between EASIX and the sFlt-1/PlGF ratio may hypothetically indicate that both markers reflect the systemic disease process in preeclampsia. While no prior study has examined the relationship between EASIX and angiogenic markers, individual components of EASIX have been linked to angiogenic imbalance. Specifically, Molvarec et al. demonstrated that the sFlt-1/PlGF ratio correlated positively with creatinine in women with preeclampsia [30]. Their correlation coefficient of 0.55 fits our observed correlation between the sFlt-1/PlGF ratio and creatinine of 0.47, supporting the generalizability of our data.

The observed moderate correlation between the sFlt-1/PlGF ratio and mean uterine PI is compatible with the hypothesis that sFlt-1/PlGF may primarily reflect uteroplacental function, consistent with the inverse albeit non-significant trend with the CPR PI observed in our cohort. This is in line with a recent randomized controlled trial by Garcia-Manau et al., who demonstrated that a management protocol based on the sFlt-1/PlGF ratio was noninferior to conventional Doppler ultrasound in avoiding adverse perinatal outcomes in small fetuses after 36 weeks of gestation [31]. Together with our findings, this may support the sFlt-1/PlGF ratio as a meaningful marker capturing information on uteroplacental and fetal status, which may explain its association with adverse perinatal events in our analysis. The AUC of 0.80 for the sFlt-1/PlGF ratio observed in our cohort is consistent with the AUC of 0.82 reported by Binder et al. in a larger, independently validated cohort, possibly suggesting the external validity of our findings [17].

In contrast, the lack of a significant correlation between EASIX and indicators of fetal status, such as CPR PI and mean uterine PI, is compatible with, but does not confirm, the hypothesis that EASIX may preferentially capture systemic maternal endothelial dysfunction, whereas the sFlt-1/PlGF ratio may reflect uteroplacental dysfunction accompanied by fetal compromise. Taken together, our findings raise the hypothesis that EASIX and the sFlt-1/PlGF ratio, while moderately correlated, capture partially distinct pathophysiological information. This is further supported by the numerically lower AUC of EASIX for perinatal adverse events in this study, in contrast to its association with maternal outcomes. This finding also refines our previous report that EASIX was independently associated with adverse maternal and perinatal events [23]. In that larger retrospective cohort, the difference in EASIX values between patients with versus without perinatal events was minimal and only marginally significant (1.05 ± 0.65 versus 0.93 ± 0.59, *p* = 0.046) [23]. Interestingly, EASIX seemed to be less influenced by gestational age in patients with preeclampsia, as described before [25]. Since sFlt-1/PlGF was strongly associated with gestational age at inclusion, which itself reflects disease severity and angiogenic imbalance, one may hypothesize that part of its numerically higher AUC for perinatal outcomes may reflect this relationship with gestational age rather than a purely biomarker-specific signal.

EASIX was associated with a shorter time to delivery, yielding a hazard ratio, both as a continuous and as a binary variable, broadly consistent in direction and approximate magnitude with our larger, independent retrospective study cohort (HR 3.00, 95% CI 1.5–6.0 vs. HR 2.25, 95% CI 1.81–2.80) [23]. This association remained significant after adjusting for gestational age at inclusion. Nevertheless, we cannot distinguish whether the observed association reflects independent biological disease progression or is partially clinician-mediated. Therefore, the observed association cannot be interpreted as evidence of independent predictive performance and should be considered exploratory and hypothesis-generating.

### Strengths and Limitations

We provide the first characterization of EASIX in correlation with the established sFlt-1/PlGF ratio in preeclampsia with direct comparison of their prognostic capacity. Several limitations of this study warrant consideration. First, this is a post hoc analysis of a prospective observational trial with a relatively small sample size. It limits the statistical power and precision of the estimates, as reflected in the wide confidence intervals. The results should therefore be regarded as hypothesis-generating rather than confirmatory, warranting confirmation in larger, independent cohorts. Second, the EASIX cut-off applied in the time-to-delivery analysis was derived from the study cohort itself and was not prespecified, introducing the risk of overfitting; external validation is required in independent cohorts before clinical implementation. Therefore, the cut-off does not represent a proposed or validated clinical threshold. Furthermore, although the calculated EASIX was not known to the treating clinicians, the individual components LDH, creatinine, and platelet count were part of routine laboratory measurements and could possibly have influenced clinical decision-making regarding timing of delivery. Third, the high proportion of early-onset preeclampsia cases may limit the generalizability of the perinatal outcome data to unselected preeclampsia populations, which are typically dominated by late-onset disease. Fourth, given the nature of a retrospective post hoc analysis, the study was not designed or powered to compare EASIX and the sFlt-1/PlGF ratio as co-primary endpoints, and the observed differences in prognostic performance should be interpreted with appropriate caution. Additionally, we acknowledge a potential incorporation bias between EASIX and the composite maternal adverse outcome, which may artificially overestimate the apparent discriminative performance of EASIX for the maternal composite endpoint. The sensitivity analysis excluding overlapping events resulted in very few cases and a lower and highly imprecise estimate. Accordingly, the point estimates for the maternal adverse composite should be interpreted with caution and should not be considered as evidence of established prognostic performance. Finally, we did not formally adjust for multiple testing, making our results hypothesis-generating rather than confirmatory.

## 5. Conclusions

This is the first study to directly compare EASIX with the sFlt-1/PlGF ratio in preeclampsia in a post hoc exploratory analysis of a prospective observational cohort. We found a moderate correlation between EASIX and the sFlt-1/PlGF ratio, and both markers were associated with a shorter time to delivery. EASIX and the sFlt-1/PlGF ratio revealed numerically similar AUC point estimates for adverse maternal events, while the sFlt-1/PlGF ratio showed a numerically higher AUC for perinatal adverse events. The observed association patterns suggest that EASIX and sFlt-1/PlGF may capture different dimensions of preeclampsia. These findings are hypothesis-generating and raise the possibility that EASIX may preferentially reflect systemic maternal endothelial activation, whereas the sFlt-1/PlGF ratio may rather reflect uteroplacental dysfunction. Our observations justify further prospective evaluation of whether combining EASIX and sFlt-1/PlGF improves risk stratification beyond either marker alone in larger, independently validated cohorts.

## Figures and Tables

**Table 1:** Baseline characteristics.

Variable	Study Cohort *n* = 32
Gestational age at study inclusion	32.5 (29.8–34.04)
Maternal age (years)	31.5 (27.3–35.8)
Pre-pregnancy body mass index (kg/m^2^)	25.7 (23.0–30.5)
Nulliparous	24 (75%)
Multiple pregnancy	7 (22%)
Gestational diabetes (dietary)	5 (16%)
Early-onset preeclampsia (diagnosis before 34 weeks of gestation)	25 (78%)
Late-onset preeclampsia (diagnosis after 34 weeks of gestation)	7 (22%)
Systolic blood pressure * (mmHg)	155 (142–162)
Diastolic blood pressure * (mmHg)	90 (78–95)
Estimated fetal weight at study inclusion	1715 (1287–2043)
Medical history	
Previous preeclampsia	2 (6%)
Diabetes mellitus type 1	3 (9%)
Chronic hypertension	6 (19%)
Antihypertensive medication	
Methyldopa	16 (50%)
Betablockers	1 (3%)
Birth characteristics	
Gestational age at delivery	33.7 (30.7–36.1)
Birth weight	1955 (1278–2455)

Data are presented as either absolute and relative numbers or median and interquartile range (IQR). * Values were assessed in a 24 h blood pressure measurement.

**Table 2:** Association between EASIX and its individual parameters with sFlt-1/PlGF ratio.

Variable	Spearman Correlation Coefficient	(95% CI)	*p* Value
EASIX	0.41	(0.07–0.67)	0.019
LDH	0.44	(0.10–0.69)	0.012
Creatinine	0.47	(0.14–0.71)	0.006
Platelet count	−0.02	(−0.37–0.34)	0.934

EASIX: Endothelial Activation and Stress Index; sFlt-1/PlGF: sFlt-1 (Soluble fms-Like Tyrosine Kinase 1)/PlGF (Placental Growth Factor) Ratio; LDH: lactate dehydrogenase.

**Table 3:** Cox proportional-hazards models for time to delivery using gestational age as the underlying time scale.

	aHR (95% CI)	*p* Value	PH Test *p* Value
Model 1:			
EASIX (continuous)	3.09 (1.08–8.84)	0.035	0.437
Gestational age at inclusion	0.95 (0.73–1.23)	0.672	0.399
Model 2:			
sFlt-1/PlGF (continuous)	1.01 (1.002–1.01)	0.003	0.987
Gestational age at inclusion	0.84 (0.64–1.09)	0.188	0.551

HR: hazard ratio per one-unit increase; CI: confidence interval; PH test *p*: *p*-value from the Schoenfeld residual test for the proportional hazards assumption (*p* > 0.05 indicates no evidence of assumption violation).

**Table 4:** Biomarker levels stratified by adverse events.

	Adverse Event Present	Adverse Event Not Present	*p* Value
Adverse maternal events			
EASIX	1.0 (0.81–1.98)	0.75 (0.55–1.05)	0.048
sFlt-1/PlGF	471.6 (173.5–604.2)	142.3 (64.6–281.1)	0.015
Adverse perinatal events			
EASIX	0.82 (0.71–1.05)	0.55 (0.47–1.21)	0.17
sFlt-1/PlGF	266.6 (153.7–535.7)	84.8 (62.2–148.2)	0.009

Data are presented as median and interquartile range (IQR) and are compared using Mann–Whitney U test.

## Data Availability

The data presented in this study are available from the corresponding author upon reasonable request.

## Supplementary Materials

The following supporting information can be downloaded at: https://www.mdpi.com/article/10.3390/diagnostics16172863/s1, Table S1: Indications for delivery; Table S2: Overview of adverse events; Table S3: Correlations between biochemical, biophysical and sonographic markers.

## Abbreviations

The following abbreviations are used in this manuscript:

EASIX	Endothelial Activation and Stress Index
LDH	Lactate Dehydrogenase
PI	Pulsatility Index
CPR	Cerebro–Placental Ratio
sFlt-1/PlGF ratio	sFlt-1 (Soluble fms-Like Tyrosine Kinase 1)/PlGF (Placental Growth Factor) ratio
MAP	Mean Arterial Pressure
AUC	Area Under the Curve
EFW	Estimated Fetal Weight
