# Supplementary material for: Association of the Endothelial Activation and Stress Index (EASIX) and the sFlt-1/PlGF Ratio in Patients with Preeclampsia
Source: Diagnostics (Basel). 2026 Sep 6;16(17):2863. doi: 10.3390/diagnostics16172863 (PMC13564489; doi:10.3390/diagnostics16172863)
Supplement: Supplementary file 1 [file diagnostics-16-02863-s001.zip › diagnostics-4492757-supplementary.pdf]

Supplemental Table S1 Indications for delivery

Adverse event	Absolute number
Pathological fetal heart rate	6
Blood pressure spike	9
Stable preeclampsia	8
Pathological doppler sonography or fetal growth restriction/arrest	6
Intrauterine fetal death	2
Prodromal symptoms	4
Hyperglycemic episode	1
HELLP syndrome	1

Multiple reasons for delivery may apply for one patient.

Supplemental Table S2 Overview of adverse events

Adverse event	Absolute number
Maternal mortality	0
Cerebrovascular events	0
Pulmonary edema	1
HELLP syndrome	2
Raised liver enzymes	5
Acute kidney injury	4
Postpartum hemorrhage	1
Placental abruption	0
Stillbirth	3
Gestational age at delivery below 34 weeks due to preeclampsia	14
Small-for-gestational age infant	19
Neonatal mortality	0
Seizures	0
Respiratory support	6
Admission to neonatal unit	8

Supplemental Table S3 Correlations between biochemical, biophysical and sonographic markers

	**EASIX**	**sFlt-1/PIGF**	**MAP**	**Mean uterine PI**	**CPR PI**
**sFlt-1/PIGF**	0.41 (0.07 – 0.67)*	1			
**MAP**	0.35 (-0.02 – 0.64)	0.25 (-0,13 – 0.57)	1		
**Mean uterine PI**	0.07 (-0.36 – 0.48)	0.53 (0.14 – 0.78)*	0.1 (-0,35 – 0.52)	1	
**CPR PI**	-0.14 (-0.51 – 0.28)	-0.37 (-0,67 – 0.03)	-0.05 (-0.46 – 0.37)	-0.23 (-0.6 – 0.23)	1
**EFW**	-0.18 (-0.51 – 0.2)	-0.6 (-0,79 – -0.30)*	-0.26 (-0.58 – 0.13)	-0.52 (-0,78 – -0.11)*	0.23 (-0.2 -0.58)

Spearman correlation coefficient with 95% CI is shown. * indicated p value <0.05. MAP: mean arterial blood pressure (*n* = 29); PI: pulsatility index; EFW: estimated fetal weight (*n* = 31) calculated according to Hadlock formula; CPR: cerebro–placental ratio (*n* = 28), calculated as the ratio of the middle cerebral artery pulsatility index to the umbilical artery pulsatility index (MCA-PI/UA-PI). Mean uterine artery PI was available for 23 patients and was measured according to the International Society of Ultrasound in Obstetrics and Gynecology (ISUOG) practice guidelines and was calculated as the average of right and left uterine artery PI.
